# Excellent response to β-1 adrenergic receptor blockade (metoprolol) and exercise restriction in high-risk type 1 long QT syndrome. A 22-year follow-up

**DOI:** 10.1016/j.hrcr.2021.12.006

**Published:** 2021-12-11

**Authors:** Williams Tejeda-Mollinedo, Sergio Díaz-Tostado, Jorge Gómez-Flores, Santiago Nava-Townsend, Moises Levinstein-Jacinto, Manlio F. Márquez

**Affiliations:** Department of Electrophysiology, Instituto Nacional de Cardiología Ignacio Chávez, Mexico City, Mexico

**Keywords:** Long QT syndrome, Metoprolol, Beta-blockers, QT interval, Syncope, Sudden cardiac death

## Introduction

Beta blockers are class I recommendation in patients with a clinical diagnosis of long QT syndrome (LQTS) to prevent sudden cardiac death.^1^ Cardiac events among patients receiving β-blocker therapy occurred in 19 of 187 (10%) LQT1 patients and 27 of 120 (23%) LQT2 patients.^2^ In a meta-analysis using a random-effect model, the use of β blocker was associated with significant risk reduction of all cardiac events (hazard ratio [HR] 0.49, *P* < .001 in 7 registry-based cohort studies [Cohort]; risk ratio 0.39, *P* < .001 in 3 interrupted time series studies) and serious cardiac events (nonfatal cardiac arrest or sudden cardiac death) (HR 0.47, *P* < .001 in Cohort), in both LQT1 and LQT2.^3^ However, different responses have been described to individual agents. For example, in the meta-analysis of Ahn and colleagues,^3^ nadolol showed a significant risk reduction in both LQT1 and LQT2 (HR 0.47 and 0.27, respectively), whereas atenolol and propranolol decreased the risk only in LQT1 (HR 0.36 and 0.46, respectively). Notably, metoprolol showed no significant reduction in either genotype. This new information seems to validate previous recommendations made by Chockalingam and colleagues,^4^ that metoprolol should not be used for symptomatic LQT1 and LQT2 patients. However, (1) nadolol is not available in all countries, and (2) as all this evidence comes from relative recent studies, there are still many patients who had been started with metoprolol and are possibly doing fine. Herein we report the case of a woman with LQT1 with excellent long-term response to metoprolol.

**Key Teaching Points**

• Most studies point out that the appropriate drug for the treatment of type 1 long QT syndrome (LQTS) is propranolol. This case shows an interesting therapeutic response to metoprolol.
• In the face of suspicion and diagnosis of LQTS, it is necessary to know if it is low, moderate, or high risk, in order to prevent cardiac arrest or sudden death.
• Other therapeutic measures such as implantable cardioverter-defibrillators may be considered if the patient does not respond to pharmacologic treatment.

## Case report

**Figure 1:**
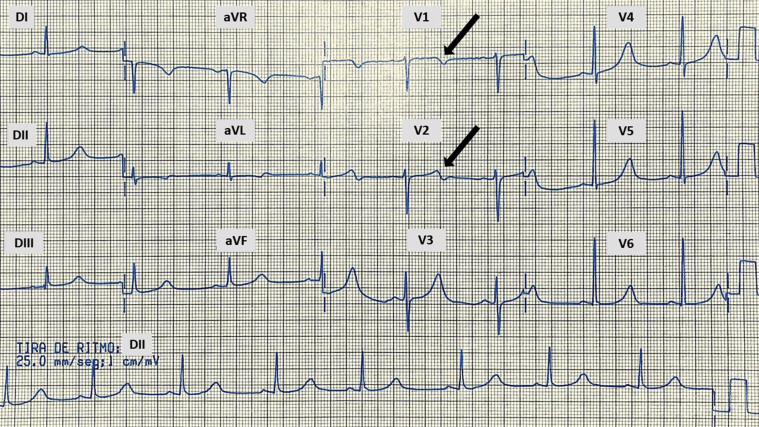
First 12-lead electrocardiogram (1999; 25 mm/s, 10 mm/mV) with QT interval of 600 ms and QTc interval of 574 ms (R-R interval 1080 ms; heart rate 55 beats/min). Notice the positive/negative T wave in leads V_1_ and V_2_ (*arrows*).

We present the case of a woman now at the age of 63 years with established clinical, electrocardiographic, and genetic diagnosis of type 1 LQTS. Her medical history is relevant for hypertension and fibromyalgia. There was no family history of sudden cardiac death. Her symptoms started at the age of 8 years when she presented episodes of sudden loss of consciousness that recurred at 9 and 10 years of age. These 3 events occurred while practicing common activities of children; all had a rapid recovery with no data of epilepsy (seizures, etc). It was not until she was 38 years old, in 1999, that she presented a witnessed nonfatal cardiac arrest while swimming; she was quickly removed from the pool and recovered spontaneously, without the need for cardiopulmonary resuscitation. She did not look for medical advice and continued swimming, but 3 months later she presented a second episode of nonfatal cardiac arrest, which required her rescue from the water, with rapid and spontaneous recovery. Neither episode had clinical semiology of epilepsy (no abnormal movements suggestive of convulsions, nor relaxation of sphincters; and the recovery of consciousness was complete and almost immediate). For that reason, she finally pursued a cardiologic evaluation. Her physical examination was normal but the electrocardiogram (ECG) showed a QTc interval of 574 ms (heart rate 55 beats/min) (Figure 1). The diagnosis of LQTS was established and it was considered as type 1 according to the morphology described by Moss and colleagues^5^ and confirmed by molecular genetic study (next-generation sequencing) that manifested a mutation in *KCNQ1*, exon 3, c.502G>A (p.Gly168Arg), heterozygous as pathogenic. She was started with metoprolol tartrate 50 mg twice a day, with excellent response and without recurrence of cardiac events since then (1999–2021, 22 years in total).

Other common cardiovascular tests were performed: Transthoracic echocardiography showed a normal left ventricular ejection fraction of 61%, mild dilation of the left atrium, and mild concentric hypertrophy of the left ventricle with associated slow relaxation and mild tricuspid regurgitation. Treadmill stress test and Holter monitoring were both normal.

**Figure 2:**
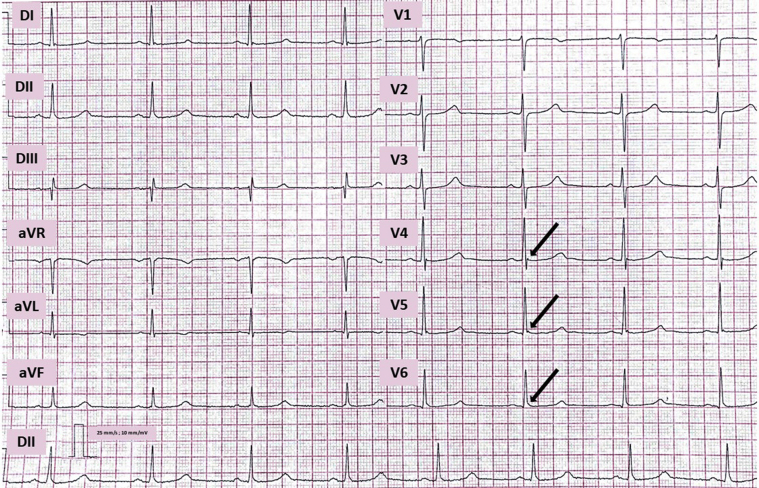
Electrocardiogram (25 mm/s, 10 mm/mV) from 2018 with a QTc by Bazett formula of 590 ms. Notice the small early repolarization pattern (*arrows*) in leads V_4_–V_6_.

**Figure 3:**
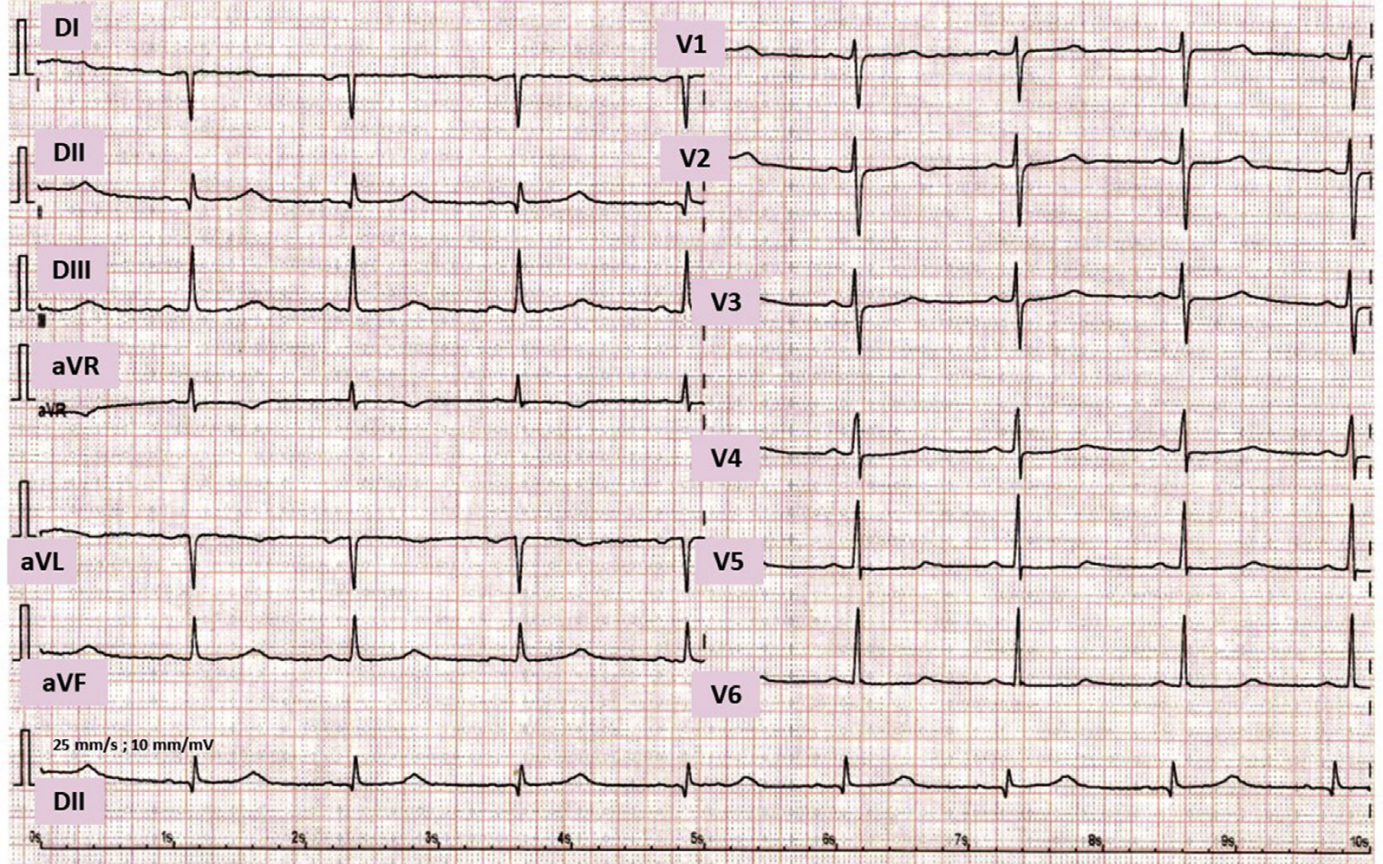
Twelve-lead electrocardiogram (25 mm/s, 10 mm/mV) and rhythm strip (DII) from 2021. QRS duration 100 ms, QT interval 554, QTc interval 500, R-R interval 1230 ms, P wave 176 ms, PR interval 198 ms.

Clinical follow-up was performed every 6 months with 12-lead ECG. Until May 2021 (last follow-up at our outpatient clinic) the patient was still asymptomatic; she has not suffered any cardiac events. The only lifestyle change was the restriction of swimming, and there was no other modification in the pharmacologic treatment during the follow-up, despite that an ECG in 2018 showed QT/QTc 612/590 ms (R-R 1240 ms; heart rate 47 beats/min) (Figure 2) and her last ECG, from May 2021, continued to show a QTc interval prolongation (500 ms by Bazett formula) (Figure 3).

## Discussion

Avoidance of genotype-specific triggers and the use of antagonists of β-1 adrenergic receptors are the basis of treatment for LQTS. Β blockers are competitive antagonists; some, such as metoprolol, are considered as “cardioselective” and others “nonselective” agents. β blockers, in general, reduce the risk of cardiovascular events by 64%^6^ and therefore they constitute the first-line treatment for LQTS, especially for LQT1. Based on the classification proposed by Priori and colleagues,^7^ the patient presented herein was classified as high risk and exercise restriction combined with metoprolol was started more than 20 years ago, when few data on the possible lack of effect of metoprolol existed.

The presence of high catecholamine state predisposes the myocardium to augmentation of transmural dispersion of repolarization, setting the stage for torsades de pointes^8^; therefore, antiadrenergic therapy is expected to be an effective modality in this group of patients. Cellular expression studies have suggested that there is a combination of decrease in basal function and altered adrenergic regulation of the IKs current in patients with C-loop missense mutations that may provide a potential explanation as to why β blockers are particularly effective in patients with this type of mutation.^9^

β-blocker therapy was associated with a significant reduction in the rate of cardiac events in probands (0.97 ± 1.42 to 0.31 ± 0.86 events per year, *P* = .001) and in affected family members (0.26 ± 0.84 to 0.15 ± 0.69 events per year, *P* = .001) during 5-year matched periods.^10^ However, differences exist in the prescription of β blockers. The main β blockers prescribed for LQTS, in series from developed countries, are propranolol in 48% and nadolol in 36%; in those series, metoprolol is only used in 3% of the cases.^11^^,^^12^

The studies behind this conduct includes the following. In 2012, Chockalingam and colleagues. studied 382 LQT1/LQT2 patients initiated on propranolol (n = 134), metoprolol (n = 147), and nadolol (n = 101) and found a higher recurrence of events under metoprolol. They explained these differences based on sodium channel blocking efficacy of propranolol compared with metoprolol, and they emphasize that symptomatic LQT1 or LQT2 should not be treated with metoprolol.

In 2017, Ahn and colleagues^3^ performed a meta-analysis and showed that atenolol significantly reduced cardiac events in LQT1 (HR 0.36, 95% CI 0.20–0.63, *I*2 = 0%, *P* for heterogeneity .33) compared to LQT2 (*P* for heterogeneity between 2 genotypes .03). Metoprolol showed a trend to decreased cardiac events only in LQT1, but there was no significant difference. In contrast, in LQT1, atenolol, propranolol, and nadolol did reduce cardiac events significantly compared with nontreated patients, whereas metoprolol did not. Again, the putative mechanism for this difference was attributed to the fact that metoprolol has minimal effects on peak or late sodium current.

Our case presentation is anecdotal evidence that metoprolol can be helpful for some patients with LQT1. Metoprolol is very likely to show efficacy in LQTS; however, it is not possible, in addition to being dangerous, to assume that it is as effective as nadolol and propranolol. Metoprolol can be considered for use in those patients who do not have an adequate tolerance to β blockers that have shown high efficacy. However, the patient was restricted from intense physical activity, so we cannot be completely sure that was only the effect of metoprolol that produced this excellent clinical result. Although actually it is recommended to use nadolol or propranolol,^12^ the relevance of this case is that, with a good response under metoprolol treatment and exercise restriction, it would be acceptable to continue with metoprolol instead of changing the drug.^13^

In LQT1, the adrenergic dependence of lethal events may fit with sympathetic activation and therefore avoidance of genotype-specific triggers for arrhythmias, as in the present case (strenuous swimming), and β-blocker use is a class I recommendation.^14^ Although in the present case it is possible that exercise restriction alone could explain the long-term survival, it can also be assumed that some benefit was provided by metoprolol. With the available evidence we cannot recommend the use of metoprolol as a first-line agent in the year 2021, but if some patient was started on metoprolol many years ago, as in the case herein presented, perhaps there is no need to change to a β blocker that requires more doses daily (propranolol) or that is not available worldwide (nadolol). Also, the use of therapies other than metoprolol may be considered first in new patients, such as left cardiac sympathetic denervation. If symptoms persist despite the use of β blockers, the use of left cardiac sympathetic denervation in LQTS has shown a significant reduction in recurrent cardiac events such as syncope or cardiac arrest, in addition to a notable shortening of the QTc.^15^

## Conclusion

A case of LQT1 with excellent response to exercise restriction and metoprolol, a cardioselective β-1 blocker, after a 22-year follow-up is presented. Perhaps patients who are doing well under metoprolol for many years do not need to be changed to another β blocker.
